# A mobile car monitoring system as a supplementary tool for air quality monitoring in urban and rural environments: the case study from Poland

**DOI:** 10.1038/s41598-023-43095-w

**Published:** 2023-09-22

**Authors:** Mikita Maslouski, Elżbieta Jarosz-Krzemińska, Paweł Jagoda, Ewa Adamiec

**Affiliations:** 1 Institute of Plant Science and Microbiology, University of Hamburg, Ohnhorststraße 18, 22609 Hamburg, Germany; 2 AGH University of Krakow, Mickiewicza 30 Av., 30-059 Kraków, Poland; 3 Krakow Smog Alert Association, Felicjanek 10/6 31-104, Kraków, Poland

**Keywords:** Atmospheric science, Environmental chemistry, Environmental impact

## Abstract

Living in healthy environment should be regarded as a primary human right and not a privilege rendered to chosen ones. For that reason, a national air monitoring grid should be as extensive as possible. Unfortunately, small towns and villages, which are also exposed to air pollution episodes are not commonly covered by monitoring grid. Fixed air monitoring systems have their limitations, which can be overcome by e.g., properly validated, reliable but cheaper mobile monitoring systems. The aim of this study was to assess the use of a car mobile PM10 monitoring system to study ambient air quality in rural communities surrounding Kraków, not covered by fixed monitoring grid. A monitoring dataset was collected during 6 winter car campaigns conducted between December 2021 and March 2022. Except for providing multiple monitoring data, the main goal pertain to methodological aspect of the mobile system, including its validation, indicating its advantages, limitations as well as providing recommendations for the future mobile studies. Our car mobile monitoring system was useful in identifying a local hot-spots with good spatial and temporal resolution, thus giving the local government decision-making tool for taking appropriate action in places not included in national monitoring grid.

## Introduction

Air pollution is proven to be one of the major health factors responsible for premature deaths worldwide^1–3^. For that reason informing communities about bad air quality episodes should be regarded as a duty not a privilege rendered only to chosen ones. Multiple research links air pollution to respiratory diseases, including chronic obstructive pulmonary disease (COPD)^4^, asthma^5,6^, lung cancer^7,8^, ischemia heart disease^9,10^, or even urinary bladder cancer^11^. Moreover, prolonged exposure to the air pollution can cause DNA damage^12^, oxidation stress and might even be associated with the brain damage, Alzheimer’s disease (AD) and other neurodegenerative disorders^13,14^. The list of negative effects of poor air quality on the human health is still growing, thus rendering this issue to be a matter of public health. Despite multiple actions conducted worldwide to improve air quality, as reported by the latest WHO data^15^, still almost 99% of the world’s population, breathes air that exceeds current World Health Organization limits. In large urban agglomerations people are usually much better informed about air pollution issues rather than the inhabitants of small towns and villages. This is mainly due to the fact that medium and large cities are most likely covered by the fixed air monitoring network, so their residents have proper access to alert and prevention response systems to smog episodes. On the other side of the spectrum there are villages and small towns where people have very limited access to the above mentioned information, which inevitably results in less awareness to air pollution issues. Therefore, there is a general misconception, especially in Poland, that air pollution prevails in large cities and avoids charming and idyllic villages and small towns, while the reality is in fact quite the opposite. IQAir 2020 Report^16^ shows that Poland is one of the most polluted countries in Europe. Among 100 cities.

with the worst air quality in the Old Continent, as many as 29 are in Poland^16^. City of Krakow, South Poland, being central point of interest in this research, for many years was ranked as top air polluted city of Europe. City’s poor air quality have improved as the consequence of ban on firing solid fuels in home furnaces introduced in 2019. However, while this prevention measure was highly anticipated by the local community, it did not ultimately resolved the problem of poor air pollution in the city of Krakow. This is due to the fact that the city is located in a valley, which favors accumulation of dust resultant from other neighboring villages and towns, which are not covered by the ban on firing fossil fuels for heating purposes. This obvious legal gap and in fact lack of centralized regulations has caused that the great effort put into reducing air pollution in Krakow was ultimately spoiled by the surrounding cities and villages. This specific situation renders Krakow to be especially interesting research site. On top of that legal issues there are other problems, related to insufficient grid of fixed monitoring around the country. Currently the National Environmental Monitoring system grid constitutes of 285 locations, including 209 automatic or automatic-manual reference stations, which provide hourly readings data shared and available online (from which there are only 135 points for automatic PM_10_ monitoring points and 45 automatic points for PM2,5 monitoring)^17^. For the second biggest Polish city of Krakow, with the total area of 327 km^2^, there are only 8 state reference fixed air quality monitoring stations (acronym SEM), while in the surrounding 13 municipalities there are only 5 stations, from which only 3 stations measure particulate matter concentration. Lack of evenly distributed and sufficient monitoring grid is not just a Polish phenomenon and this situation pertains to many countries in the world. Filling in the gaps in data provided by stationary fixed monitoring systems with e.g. validated mobile systems may, in a much more personalized and flexible way, be useful in mapping air pollution episodes in places (rural and/or urban) so far not included in monitoring grid. There is no ideal ambient air monitoring system. Each system has its own limitations or simply serves a different purpose. Fixed stationary air monitoring systems, based on gravimetric methods of measurements are without a doubt the most accurate system with low uncertainties errors, but are also very expensive, highly engineered systems. They allow to monitor air quality changes over time^18^ but due to cost barrier a number of installed units are most commonly limited thus an air pollution data set is not obtained from the entire city. On the other hand, a mobile measurements are especially useful in identifying hotspot and the point sources of pollution in real-time conditions providing multipollutant data in function of time and location^19^. These mobile systems contain commonly a low to middle-cost sensors, which are definitely much more budget friendly solutions and more likely installed in various rural locations, not included in national monitoring network. However, lower price of the sensors may come with less accuracy of the obtained dataset when compared to a reference measuring station. This is due to the fact that the method of measurement, which is based on light scattering is much more affected by meteorological conditions^20,21^ and requires proper calibration and validation of the apparatus. Moreover, as indicated by e.g. Samad and Vogt^22^ while stationary monitoring stations provide high temporal resolution of a specific location they provide very limited information on spatial distribution of the monitored parameters. It seems therefore a reasonable approach to combine the stationary and mobile systems to achieve the most accurate results and deliver larger data set. Researchers suggest that the best option is to adjust the mobile units based on the value of fixed local reference station^23^ or to combine mobile and stationary air pollution monitoring with neural network models^24^. An approach of using joint mobile and stationary systems is growing in the field of urban air pollution monitoring. There are several ongoing studies on mobile air monitoring conducted worldwide. For instance, Samad and Vogt^22^ were employing bicycle (MOBAIR) measuring station, equipped with aerosol spectrometer, aethalometer, GPS camera, weather station, NO_2_/NO, NOx monitors, ozone meter and finally condensate particle counter to investigate air quality in German cities. This mobile platform was found to be successful in providing reliable as well as user friendly measurements and was considered as highly adaptive and flexible method for air quality monitoring. Other successful study was conducted on German–Czech border^25^ using mobile trolley stations, which were proven to be suitable in effective recording of spatial and temporal characteristics of air quality. Adams and Corr^18^ has obtained extensive air monitoring dataset collected for total of 11 years using a mobile platform installed in a van with air intake located on the roof. Authors have then formulated multiple useful recommendations for performing proper mobile collection data, such as “drive and park approach”, regular monitoring schedule, conducting a test for mobile vehicle self-pollution and many others. Mobile air quality measurements were also carried out in Poland, conducted by the Polish Smog Alert Association—an anti-smog movement united as local civic initiatives (such as Krakow Smog Alert Association) to improve air quality at the local and national level. The aim of their mobile research conducted in 2016 by Bartyzel^26^ was to make people in smaller towns aware that the problem of air pollution does not only pertain to large cities but, on the contrary, villages and small towns, where domestic heat sources mainly contribute to PM emissions in amounts exceeding greatly WHO standards. Bartyzel et al.^26^ in their research have determined an appropriate conditions to perform such a mobile measurement. They have found and scientifically proved that the late evening/night measurements were much more justified than the daytime measurements due to several factors, including limiting the impact of car traffic (another source of PM_10_ dust) and most of all the stability of weather conditions. Authors have pointed out that the temperature inversions during nighttime measurements are reducing the vertical mixing of the atmosphere. In their mobile air quality measurements Authors had paid a special attention to the correct location of the inlet, the air intake points on the mobile station in relation to the vehicle geometry, which in fact have determined much better quality of the obtained data. The correct location of the inlet have greatly limited the dependence of the measured concentrations on the vehicle speed. It was found that in order to determine the best quality results a car speed during mobile measurements should not exceed 40 km/h and the inlet should be located parallel to the driving direction. This is therefore different approach than for instance one proposed by Adams and Corr^18^, to situate an air intake centered on the vehicle roof.

The aim of our research study was to assess ambient air quality using the car mobile PM_10_ monitoring system in rural communities surrounding Krakow, excluded from fixed monitoring grid and not included in a ban on burning coal in households introduced in 2019 in Krakow. Except for obvious purpose of providing multiple monitoring data including local hot-spots with the function of time and location, the main goal of our study pertains to methodological aspect of the used system, including its validation as well as indicating its advantages and drawbacks. The limitations and recommendation for conducting mobile air monitoring research were then be formulated for the future similar research.

## Materials and methods

### Study area

**Figure 1:**
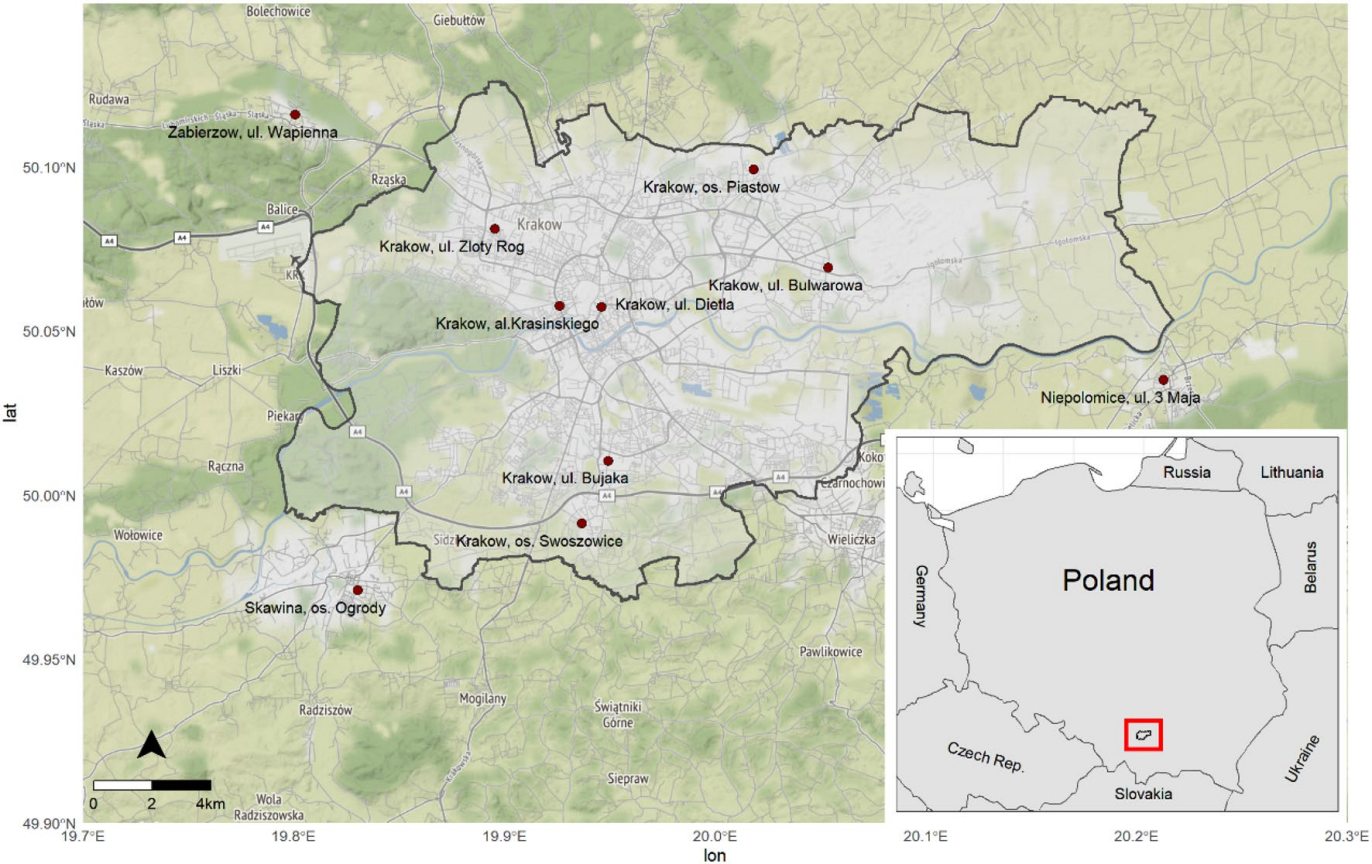
Study area including state environmental monitoring grid (SEM) reference stations marked as red points.

An ambient air quality (PM_10_ concentration) was studied during six mobile sampling campaigns conducted in winter months between December 2021–March 2022 in rural cities/villages surrounding Krakow, that is Węgrzce, Przybyslawice, Niepolomice i Libertow. Each sampling route was initiated and ended at the very same point that is in vicinity to the Faculty of Physics AGH UST in Kraków. All mobile measurements routs (referred as transects) were planned in proximity to State Environmental Monitoring stations (SEM), being stationary, fixed reference stations owned by the Chief Inspector of Environmental Protection. Total travel distance covered during all measurement campaigns included area of 653 km. Figure 1 depicts study area, including administrative boarders Krakow and state environmental monitoring grid (SEM) stations.

### Methods

**Figure 2:**
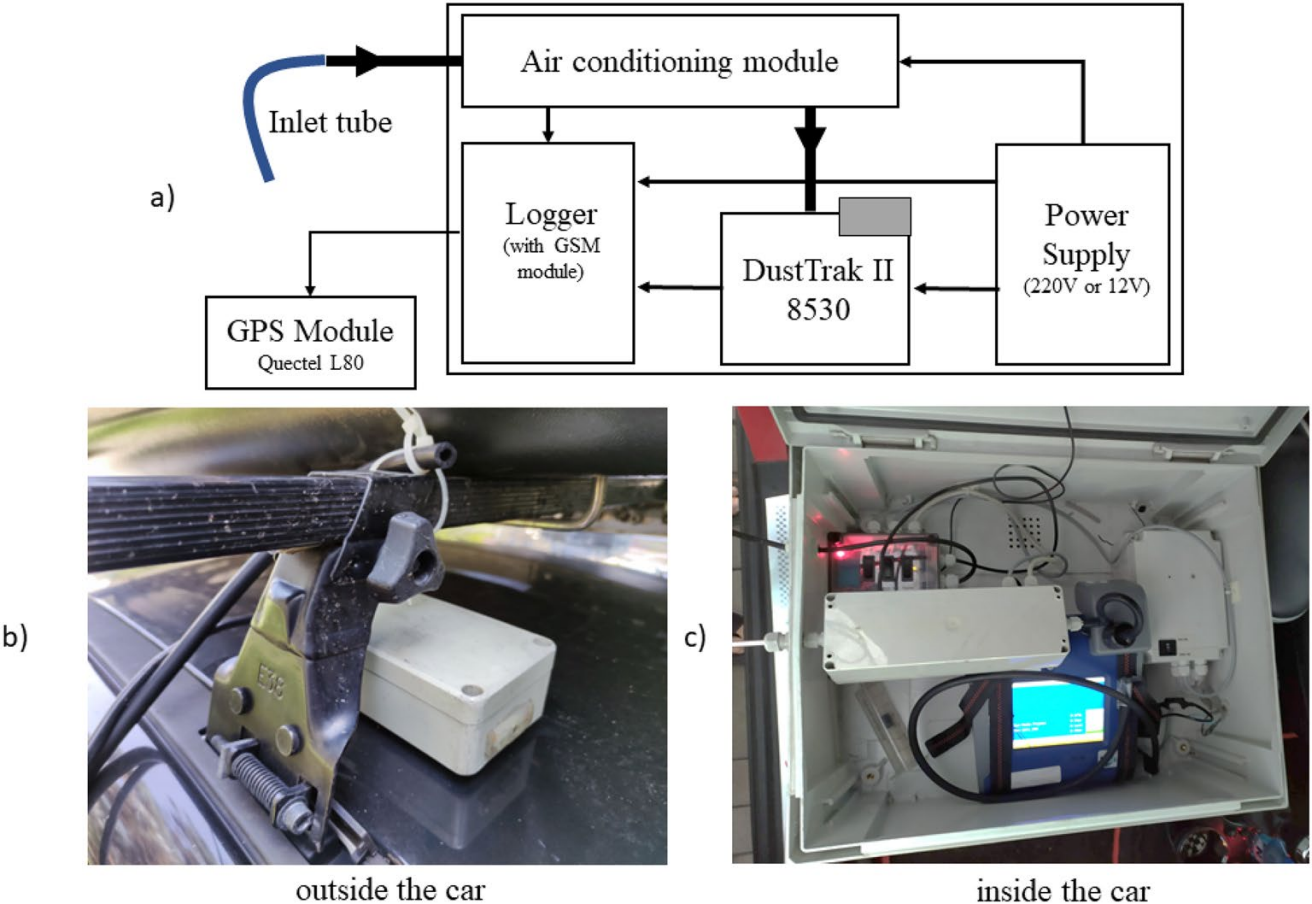
Mobile air monitoring station scheme (**a**) air inlet and GPS module location (outside the car, through the front passenger seat window) (**b**) panel (**c**) shows arrangement of the instruments.

The mobile air monitoring unit used in this study consisted of the DustTrak II Aerosol Monitor 8530 equipped with the following modules: a module for data transmission and logging, GPS antenna with a magnetic mount, a module for air conditioning prior measurement and the power supply that enables the device to be powered with 12 V or 220 V. The air conditioning module consisted of the stainless steel tube, which was heating the sampling air to the temperature above 100 °C to prevent formation of water droplets. Details regarding the mobile monitoring station scheme is depicted on Fig. 2.

A mobile monitoring station used in the study is a property of the Krakow Smog Alert Association (acronym KAS), which was borrowed for the purpose of this in-situ research.

Suspended dust particles PM_10_ were recorded by the measuring devices at a frequency of 3 Hz, in accordance with the measurement methodology proposed by Bartyzel et al.^26^. The station was located on the passenger seat, the GPS module and the inlet were placed outside the car through the passenger seat window. The power was supplied via a suitable converter to the cigarette lighter socket.

The measurements were carried out during the night hours between 8 PM and 2 AM, when the boundary layer is thinnest due to inversion, thus creating stable meteorological conditions. The lack of intense car traffic at this time of the day allows to associate a single hot-spots with local sources of dust pollution (referred as “low emission”, released by emitters up to 40 m high, mainly from domestic boilers). Car driving speed was not exceeding 40 km/h.

Table 1 describes a log on mobile measurement routes (transects), including meteorological parameters during 6 sampling campaigns. Single mobile measurement route distance was about 50–170 km, measurements were lasting approximately 2–5 h, an average car speed was approx. 35 km/h. Meteorological parameters provided in Table 1 were obtained from Krakow-Balice airport station located in the North-East Krakow.

**Table 1:** Routes summaries with meteorological conditions.

Transect*	Date	No SEM stations	Distance (km)	Mean car speed (km/h)	Ambient temp (°C)	Humidity (%)	Pressure (hPa)	Wind speed (m/s)	Wind direction** (°)
1	2021-12-30	3	49	29.6	4.6 ± 0.6	94.7 ± 2.0	1017.4 ± 0.2	2.7 ± 0.5	217.3 ± 2.5
2	2022-01-13	5	85	30.4	3.1 ± 0.2	84.7 ± 5.5	1031.1 ± 0.5	6.7 ± 1.0	234.7 ± 3.0
3	2022-01-19	6	102	37.1	0.8 ± 2.2	81.3 ± 3.5	1016.4 ± 0.8	4.8 ± 3.5	216 ± 13
4	2022-02-13	5	104	33.2	5.2 ± 0.9	79.5 ± 5.0	1020.9 ± 0.8	2.3 ± 1.0	20 ± 11
5	2022-02-15	8	143	38.6	0.4 ± 0.3	92.0 ± 1.0	1016.2 ± 0.7	0.8 ± 0.5	180 ± 170
6	2022-03-03	7	170	37.2	0.8 ± 0.3	75.5 ± 3.0	1019.4 ± 0.2	1.8 ± 1.0	260 ± 180

*Transect: 1, Przebyslawice → Wegrzce; 2, Libertow → Wieliczka → Niepolomice; 3, Wegrzce → Przebyslawice → Zabierzow → Skawina → Libertow; 4, Wegrzce → Przebyslawice → Zabierzow → Skawina → Libertow; 5, Wegrzce → Przebyslawice → Zabierzow → Skawina → Libertow → Wieliczka → Niepolomice; 6, Libertow → Wieliczka → Niepolomice → Wegrzce → Przebyslawice.

**0° represents North; 90°—East; 180°—South; 270°—West.

All routes have the same staring and end point location- Reymonta Street 19 (Faculty of Physics and Applied Computer Science, AGH UST).

### System calibration and the validation of the obtained results

The mobile monitoring station was calibrated prior conducting our in-situ research with the stationary reference stations (SEM) in order to obtain high quality results. Moreover an additional in-situ calibration was performed according to manufacturer’s recommendations, in order to adjust the calibration coefficients to the local conditions.

In-situ calibration of the mobile station was conducted in the town of Skala, approx. 20 km in the North of Krakow. The calibration process lasted about 23 days during November 2021, when over 500 samples were recorded as hourly data readings in a wide range of values—from 20 µg/m^3^ to even 800 µg/m^3^. The Pearson correlation coefficient was equal R = 0.89 (p-value ≪ 0.05). Additionally, multiple zero tests have been performed throughout the campaign.

Furthermore, additional online databases from the fixed reference air quality stations (SEM)^27^ were used in order to analyze and interpret the obtained results.

### Statistical significance of the obtain readings

PM_10_ readings from mobile the DustTrak station were averaged within 50 m from the fixed, reference stations (SEM). Measurements were then compared with the SEM station readings. However, what is especially important is the fact that the Dustrak’s reading are averaged to 1 min when the fixed reference station provides data as hourly reading. Therefore, we have assumed, that the measurements (readings) obtained from both the mobile and fixed stations can be compared only when the state of the atmosphere is close to stable, according to the Pasquill scale. In such case, the hourly average dust concentration changes only in small ranges. The ratio of an hourly average PM_10_ concentration obtained from the fixed reference station to our Dustrack minute average reading was calculated. Furthermore, the mean value ($\overline{{\text{x}}}$) and the standard deviation (σ) of rations were then determined. Only the results from the stations in the range $\overline{{\text{x}}}$ ± σ were considered for farther analysis. The rejection of the extreme ratios—beyond the $\overline{{\text{x}}}$ ± σ range—is supported by the fact that the dust meter during the measurement near the station could measure a temporary but significant change in dust concentration, which will be visible on a scale of several minutes, but not on hourly scale (e.g. making impossible to detect e.g. hot spot episodes caused by fast-burning rubbish in the fireplace).

Furthermore, to define the statistical relevance of the mobile measurements, the Pearson and Spearman regression coefficient for the DustTrak and SEM stations were calculated (Table 2). It is assumed that for high (R > 0.5) correlations, the measurement can be considered as statistically representative.

**Table 2:** Statistical significance of the obtained readings from both mobile and stationary SEM reference stations.

Date	Reference station location	Reference station readings		Car DustTrak readings	
		Time	PM_10_ (µg/m^3^)	Time	PM_10_ (µg/m^3^)
2021-12-30	SEM Krakow, Zloty Rog	21:00	41.6	20:04	37.62
	SEM Krakow, Dietla*	22:00	53.2	21:43	39.51
	SEM, Krakow Krasińskiego	22:00	38.7	21:53	39.79
	**Statistic (test result)**	**Pearson**	**–**	**Spearman**	**-**
2022-01-13	SEM, Krakow Krasińskiego	21:00	27.1	20:36	18.29
	SEM, Bujaka*	21:00	29.1	20:53	13.66
	SEM, Lusińska	22:00	21.0	21:28	14.58
	SEM, Niepolomice	23:00	20.5	22:19	16.12
	SEM, Krakow, Bulwarowa	23:00	26.1	22:46	16.72
	**Statistic (test result)**	**Pearson**	**0.83**	**Spearman**	**0.8**
2022-01-19	SEM Krakow, Zloty Rog	22:00	88.3	21:53	92.38
	SEM Zabierzow, Wapienna*	23:00	119.5	22:56	67.83
	SEM Skawina, os. Ogrody	00:00	85.3	23:49	98.19
	SEM, Krakow, Lusińska	01:00	57.9	00:14	81.17
	SEM, Krakow Bujaka	01:00	85.6	00:21	94.12
	SEM, Krakow Krasińskiego	01:00	77.0	00:30	66.89
	**Statistic (test result)**	**Pearson**	**0.52**	**Spearman**	**0.5**
2022-02-13	SEM Krakow, Zloty Rog	23:00	62.0	22:21	48.29
	SEM Zabierzow, Wapienna	00:00	77.4	23:29	92.51
	SEM Skawina, os. Ogrody	01:00	56.1	00:15	78.21
	SEM, Krakow Bujaka	01:00	39.3	00:55	48.31
	SEM, Krakow Krasińskiego	02:00	61.7	01:05	66.70
	**Statistic (test result)**	**Pearson**	**0.92**	**Spearman**	**0.8**
2022-02-15	SEM Krakow, Zloty Rog*	22:00	130.1	21:03	37.11
	SEM Zabierzow, Wapienna	23:00	136.2	22:10	86.79
	SEM Skawina, os. Ogrody	23:00	78.3	22:49	63.38
	SEM, Krakow, Lusińska	00:00	89.3	23:08	63.31
	SEM, Niepolomice	00:00	122.8	23:43	92.74
	SEM, Krakow, Bulwarowa	01:00	96.9	00:08	54.64
	SEM, Krakow, Dietla*	01:00	54.7	00:22	70.70
	SEM, Krakow Krasińskiego	01:00	117.0	00:27	65.89
	**Statistic (test result)**	**Pearson**	**0.77**	**Spearman**	**0.71**
2022-03-03	SEM, Krakow Krasińskiego*	22:00	84.6	21:17	57.08
	SEM, Niepolomice	00:00	68.4	23:32	58.29
	SEM, Krakow, Bulwarowa	01:00	45.1	00:06	44.72
	SEM, Krakow, Dietla*	03:00	17.5	02:07	30.45
	SEM, Krakow Krasińskiego	03:00	36.6	02:14	29.53
	**Statistic (test result)**	**Pearson**	**0.96**	**Spearman**	**0.99**

*Data beyond the $\overline{{\text{x}}}$ ± σ range.

Statistic test results are in bold.

Measurements outside the $\overline{{\text{x}}}$ ± σ interval were not included for farther analysis, marked in gray and asterisked in the Table 2.

The car mobile monitoring provides temporary data, being representative only of a short period of time is compared to stationary measurement of hourly average. Despite this discrepancy the results from the stations presented in Table 2 revealed good statistical agreements. Pearson’s and Spearman’s correlation coefficient (R) was above 0.5, which proves significant correlation and thus statistical significance of data obtained during 5 out of 6 measuring days.

## Results and discussion

**Figure 3:**
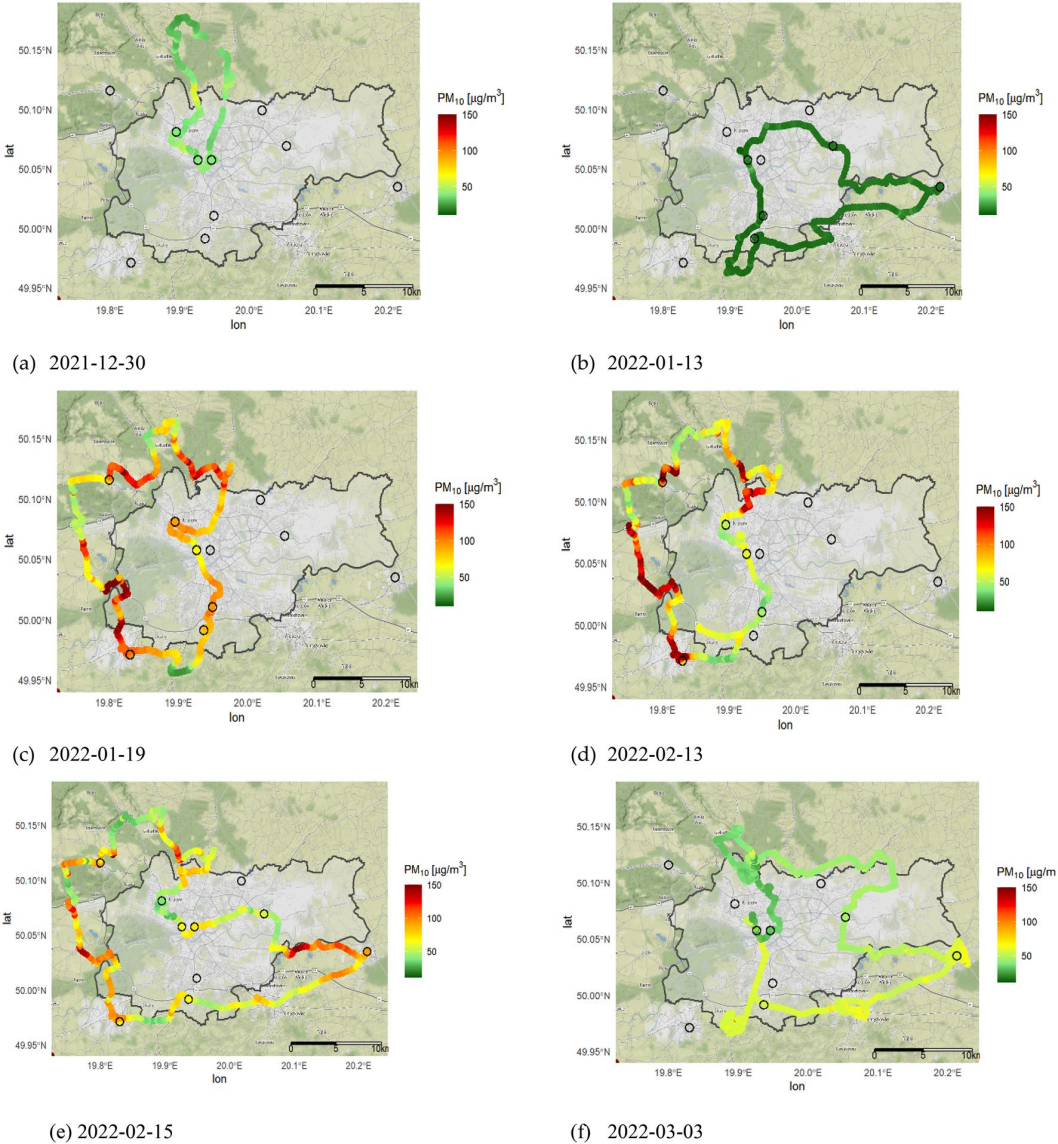
Mobile transects obtained in winter 2021–2022. The SEM reference stations marked as black circles.

A graphical presentation (maps of transects) of all in-situ mobile dataset (depicted on Fig. 3) were plotted with the software R software (ggmap package). Krakow administrative boundaries were obtained from GAMD database.

Each point depicted on maps represents the average value of PM_10_ concentration recorded by the dust meter in 20 s with a frequency of up to 3 Hz (approx. 40–60 readings). In addition, samples collected in the exact same location (for example during stops next to the reference stations) were averaged to one reading.

All transects were obtained during evening and night hours (8:00 p.m.–2:30 a.m.), when the atmospheric conditions were considered as stable (transect presented on Fig. 3b,c) or slightly stable (remaining transects), according to the Pasquill Stability Classes.

All transects except for the first one (Fig. 3a) were considered as statistically significant, since the Pearson and Spearman coefficients were found to be above 0.5. The first transect was then rejected due to an insufficient number of reference stations necessary for the calculation of statistical coefficients.

In the analysis we have also considered the changes in meteorological parameters, well known to influence the PM_10_ concentration in ambient air, since the main functionality of our mobile monitoring system was to determine the hot spot occurrence in the villages and cities surrounding Krakow. Danek et al.^20^ for instance indicate that the probability of “low” emission (explained earlier as emission up to 40 m high, derived mainly from domestic households and car traffic) rises when the ambient air temperature is close or less than 0 °C; air humidity is above 70%; and the wind speed is low. Moreover, during long term research carried out by KAS, it was concluded that the „low” emissions is directly correlated with the hours of human activity in the place of residence, that is in the morning (approx. 6:00–9:00 a.m.) and in the evening (5:00 p.m.–1:00 a.m.). All above mentioned factors were considered during our mobile monitoring campaigns.

When analyzing all individual monitoring campaigns it was found that the lowest concentrations of PM_10_ concentration ranging from 7 to 31 µg/m^3^ were recorded in January 13th on Thursday evening, when the relative humidity was approximately 84.7 ± 5.5%, SW wind direction and ambient temperature of about 3.1 ± 0.2 °C. Transect depicted on Fig. 3b was recorded in the appropriate time interval (between 20:00–23:00). However, since the wind speed on January 13th was much more intensive when compared to other campaigns weather conditions, this has probably contributed to the reduction of PM_10_ concentration. The average PM_10_ dust concentration that night was close to regional background value. Surprisingly, when considering other meteorological parameters such as ambient temperature it was found that the highest 20 s averaged PM_10_ concentration (approximately 225 µg/m^3^) were recorded in February 13th when the outside air temperature was the highest from all 6 sampling campaigns, being above 5 °C.

Transect presented on Fig. 3f, was conducted on March 3rd 2022 in slightly stable weather conditions. The beginning of the transect was mainly marked as yellow, which refers to an elevated PM_10_ concentrations above 50 µg/m^3^ with a single hot spot found in the village of Libertów and on the route to Wieliczka (both places located on the south side from the center of Krakow), where PM_10_ concentration was recorded as high as 174 µg/m^3^. Towards the end of the measurement the north side of Krakow in the city of Niepołomice gradual purification of ambient air was observed, and the PM_10_ concentration decreased approximately from 70 to 40 µg/m^3^ (presented as green scale).This phenomena is also visible in SEM dataset, which shows over 80 µg/m^3^ in the city center at 22:00 and below 40 µg/m^3^ at 02:00.

Much more diverse PM_10_ concentrations were recorded during campaign conducted on January 19th, February 13th and February 15th 2022 (results are depicted on Fig. 3c–e). The transects revealed big difference in PM_10_ concentration in the Krakow city center and its borders. The rural areas (villages) North and West of the city center deserve special attention, as there is a sharp shift from low PM_10_ dust concentration 23–30 µg/m^3^ to as high concentration as 176–225 µg/m^3^.

**Figure 4:**
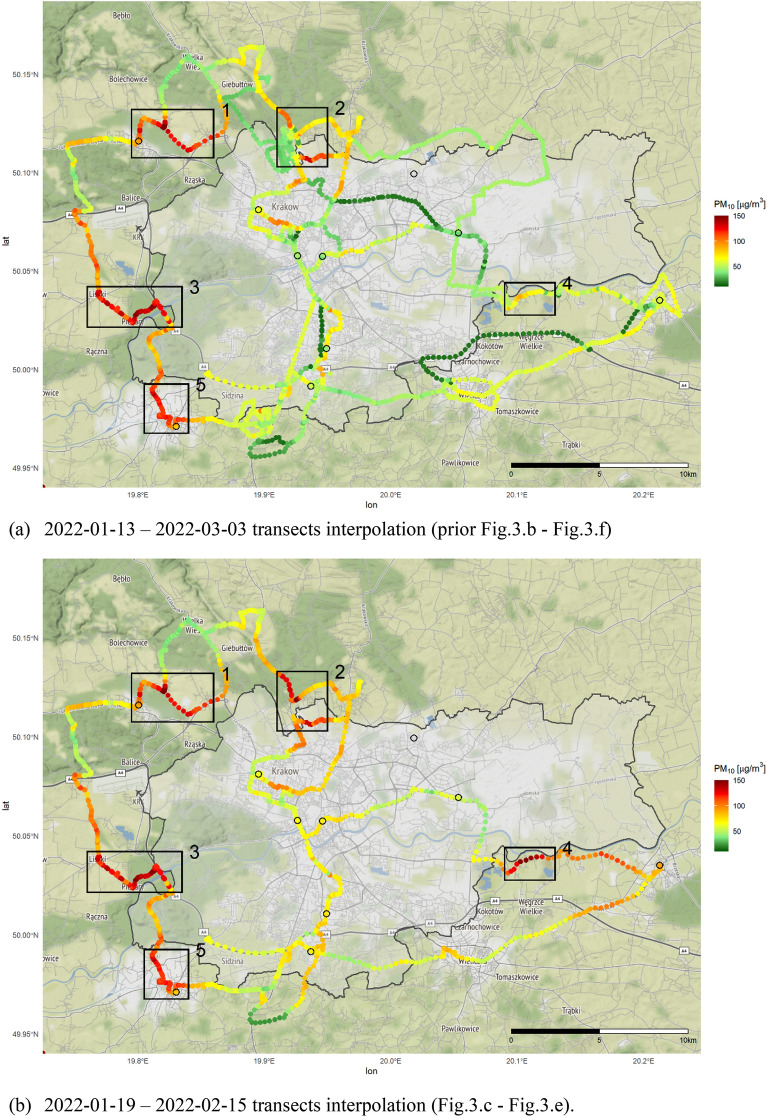
Transect averaged to one map (**a**) and for the selected measurements campaigns (**b**).

All transects were then combined to one single map. Figure 4a depicts all the readings (Fig. 3b–f) while Fig. 4b summarizes only 3 selected campaigns’ transects previously presented on Fig. 3c–e. Values of PM_10_ concentration in Fig. 4 represents the PM_10_ averaged value for a given point obtained within a radius of 150 m. Rapid changes in dust concentrations have been marked and numbered. Administrative borders of Krakow city also marked on the maps.

The increased concentration of PM_10_ was recurrently recorded in the west boarder of Kraków, in places marked as 1 (the city of Zabierzów), 3 (the Vistula riverbed, villages of Liszki and Piekary), and 5 (the city of Skawina). A repeated episodes of high PM_10_ dust concentration being recorded in the very same locations proves them to be a local „low” emission hot spots in the vicinity of Krakow. Point marked as 3 on Fig. 2 requires an additional study, as it is located next to the Vistula riverbed—a place where local pollutants can accumulate. The highest concentrations of PM_10_ reaching up to 250 µg/m^3^ were recorded repeatedly during the entire campaign, in all sampling location 1 (Zabierzów), 2 Zielonki, 3 (Liszki), 4 (Zabierzów and Wieliczka) as well as 5 (Skawina). Above mentioned „low” emissions hot spots coincides with the largest number of solid fossil fuel furnaces, which have not yet been replaced in these municipalities as part of Voivodeship air protection programs. According to data provided by Stowarzyszenie Metropolia Krakowska^28^ by the end of 2021 there were still 19,315 furnaces to be replaced in rural municipalities in proximity to Krakow borders. Without proper and centralized measures these municipalities will still remain being responsible for secondary air pollution load for the Kraków city.

## Conclusions

A mobile air monitoring system serves different purpose than the stationary fixed monitoring system. For that reason it should be considered as a supplementary tool extending air quality monitoring grid. Our research revealed that properly validated car mobile monitoring system can become a useful tool in providing good quality data obtained for much more extended study area than ever possible with the use of stationary grid. This highly adaptive and flexible tool when properly validated can provide good quality data, including spatial distribution of multiple air pollutants and local hotspots The dataset obtained from the mobile monitoring may become useful for farther numerical or simulation studies or just become a decision making tool for local government. Mobile monitoring system can for instance provide locals with the information which cannot be otherwise obtained from the fixed monitoring stations (based on hourly data readings), such as suspicious activities including illegal burning rubbish in home furnaces. Moreover, mobile monitoring data may be useful in estimating the amount of pollution load entering Krakow from a certain location, thus allowing more conscious decision-making processes for local government. This tool can finally be useful in building public awareness of an air pollution problem in places not covered by fixed monitoring grid. However, at the same time a dataset derived from our mobile system cannot be used solely to draw appropriate conclusions on formation and the spread of pollutants in the study area. This is due to the fact that the quality of the obtained results strictly rely on data provided by a stationary fixed measurement. Mobile measurements should be therefore considered as a complementary tool to fixed stationary monitoring systems.

The outcomes of our research allowed to formulate general recommendations for other future car monitoring PM_10_ studies:

1. Before initiating data collection, the mobile monitoring units should be additionally calibrated to local conditions.
2. Data readings should be initiated and ended in the proximity to the fixed reference stations, using drive and park approach (lasting approximately 15 min). It is then recommended to determine statistical significance of the monitoring readings by comparing dust meter readings obtained in the vicinity of the fixed station (according to the description in point 2.3).
3. When assessing ambient air quality related to everyday life (derived mostly from households), the readings should be collected after dark (between 21:00 p.m. and 01:00 a.m.), in stable atmospheric conditions, when other sources of pollution, including traffic related air pollution, are limited.
4. Vehicle speed during mobile measurements should not exceed 40 km/h in order not to disturb the reading of the station.
5. Mobile vehicle unit should be marked with a special stickers and yellow warning signals since it will be moving with a low driving speed.
6. Each mobile measurement results should be interpreted in the context of local demographics and geographical conditions.

### Supplementary Information

Supplementary Information 1.

Supplementary Information 2.

Supplementary Information 3.

Supplementary Information 4.

Supplementary Information 5.

Supplementary Information 6.

Supplementary Information 7.

## Data Availability

The meteorological datasets analyzed in the current study are publicly available on the following website: https://danepubliczne.imgw.pl/data/dane_pomiarowo_obserwacyjne/dane_meteorologiczne/terminowe/synop/2022/. Moreover, datasets obtained from the fixed reference air quality stations, which were used in the current study are publicly available and can be accessed via https://powietrze.gios.gov.pl/pjp/maps/measuringstation/Z. The raw datasets that support the findings of this study are included as a supplementary files.
